# MOVING TOGETHER: IMPACT OF LIVESTREAM GROUP PROGRAM FOR PEOPLE LIVING WITH COGNITIVE IMPAIRMENT AND CARE PARTNERS

**DOI:** 10.1093/geroni/igad104.0427

**Published:** 2023-12-21

**Authors:** Deborah Barnes, Jennifer Lee, Margaret Chesney, Wolf Mehling, Francesca Nicosia, Rebecca Sudore, Linda Chao, Cynthia Benjamin

**Affiliations:** University of California, San Francisco, San Francisco, California, United States; Together Senior Health, San Francisco, California, United States; University of California, San Francisco, San Francisco, California, United States; University of California, San Francisco, San Francisco, California, United States; University of California, San Francisco, San Francisco, California, United States; University of California, San Francisco, San Francisco, California, United States; University of California, San Francisco, San Francisco, California, United States; Together Senior Health, San Francisco, California, United States

## Abstract

Moving Together is an online, mind-body, group movement program for people living with cognitive impairment or mild dementia (PLCI/D) and care partners (CPs). We performed a randomized, controlled trial to test the impact of Moving Together on quality of life (primary) or physical, cognitive, social or emotional (secondary) outcomes in PLCI/D or CPs. Study participants were enrolled as dyads and randomly assigned to intervention or waitlist control groups. Participants joined live-streaming classes for 1 hour, 2 days/week, for 12 weeks. Intention-to-treat analyses compared 12-week change between groups. We assessed 603 dyads for eligibility: 382 (63%) declined to participate, primarily due to lack of interest; 123 (20%) were ineligible, primarily due to lack of CP or moderate/severe dementia; and 98 (16%) were eligible and enrolled. Of these, 77 (79%) completed the 12-week assessment and 21 (21%) withdrew, primarily due to health or scheduling issues. PLCI/D were 77±11 years old, 44% women, 22% non-White/Hispanic, 32% Alzheimer’s disease, 26% unspecified/mixed dementia, 14% mild cognitive impairment, 10% vascular dementia, and 19% other/unknown causes. CPs were 66±12 years old, 78% women, 31% non-White/Hispanic, 71% spouses, 25% adult children, and 3% paid. Baseline characteristics were similar between groups. Participants who completed the program reported high satisfaction (98% good/excellent). There were no serious adverse events related to study participation. We conclude that Moving Together is a safe program with good retention and high satisfaction. Data collection will be completed in May 2023, and we will present efficacy outcomes at the meeting.

